# Proton Magnetic Resonance Spectroscopy Characterization of Rathke’s Cleft Cysts (RCCs): Relevance to the Differential Diagnosis of Pituitary Adenomas and RCCs

**DOI:** 10.3390/cancers12020360

**Published:** 2020-02-04

**Authors:** Omkar B. Ijare, Martyn A. Sharpe, David S. Baskin, Kumar Pichumani

**Affiliations:** 1 Kenneth R. Peak Brain and Pituitary Tumor Treatment Center, Department of Neurosurgery, Houston Methodist Neurological Institute, Houston Methodist Hospital and Research Institute, Houston, TX 77030, USA; oijare@houstonmethodist.org (O.B.I.); masharpe@houstonmethodist.org (M.A.S.); 2 Department of Neurological Surgery, Weill Cornell Medical College, New York, NY 10065, USA

**Keywords:** Rathke’s cleft cyst, pituitary adenoma, magnetic resonance spectroscopy, glycosaminoglycans, cholesterol

## Abstract

Background: Rathke’s Cleft Cysts (RCCs) are rare epithelial cysts arising from remnants of the Rathke pouch in the pituitary gland. A subset of these lesions enlarge and produce a mass effect with consequent hypopituitarism, and may result in visual loss. Moreover, some RCCs with a high intra-cystic protein content may mimic cystic pituitary adenoma, which makes their differential diagnosis ambiguous. Currently, medical professionals have no definitive way to distinguish RCCs from pituitary adenomas. Therefore, preoperative confirmation of RCCs would be of help to medical professionals for the management and proper surgical decision making. The goal of this study is to identify molecular markers in RCCs. Methods: We characterized aqueous and chloroform extracts of surgically resected RCCs and pituitary adenomas using ex vivo ^1^H NMR spectroscopy. Results: All RCCs exclusively showed the presence of mucopolysaccharides which are glycosaminoglycans (GAGs) made up of disaccharides of aminosugars and uronic sugars. Conclusion: GAGs can be used as metabolite marker for the detection of RCCs and this knowledge will lay the groundwork for the development of a non-invasive, in vivo magnetic resonance spectroscopy methodology for the differential diagnosis of RCCs and pituitary adenomas using clinical MRI scanners.

## 1. Introduction

Rathke’s cleft cysts (RCCs) are rare benign epithelial cysts arising from remnants of the Rathke pouch in the pituitary gland [1]. Histologically, RCCs are composed of well-differentiated, ciliated columnar epithelia which are made up of a single ciliated or stratified ciliated columnar cell linings [2]. RCCs are often discovered incidentally during an autopsy [1]. Given RCCs are rare pituitary cysts, the prevalence of these lesions is unclear. However, in a series of 1000 postmortems, RCCs were found in 113 cases (11.3%) [1]. Nowadays, magnetic resonance imaging (MRI) has become an indispensable neuroimaging tool and thus helped in the diagnosis of RCCs as well as other sellar and suprasellar tumors [3]. The vast majority of these cysts are asymptomatic, but if they are large enough and exert pressure on the surrounding structures such as optic chiasm, they may cause visual disturbances, headache, and pituitary dysfunction [1]. A subset of these lesions enlarge either slowly or, occasionally, rapidly due to hemorrhage, and produce a mass effect with consequent hypopituitarism and at times, visual loss either due to slow expansion or sudden hemorrhage. Moreover, epithelial stratification in RCCs is caused by inflammation that can extend into the adjacent adenohypophysis or neurohypophysis which may result in panhypopituitarism [2]. RCCs are more common in adults, with a peak incidence in the age range 30–50 years [1], while pediatric cases are very rare [4]. Although MRI can be highly suggestive of this diagnosis, a definitive clinical diagnosis cannot be made from imaging alone [5,6,7]. The signal intensity of MRI images of RCCs is variable with some showing hyper-intensity on T_1_-weighted images, while others appear brighter on T_2_-weighted images [5,6]. A cyst containing a clear fluid like cerebrospinal fluid would appear hypointense on T_1_-weighted and hyperintense on T_2_-weighted imaging, whereas a cyst with thick, mucoid contents would appear hyperintense on T_1_-weighted and isointense on T_2_-weighted imaging. On the other hand, a cyst with blood would be hyperintense on both T_1_- and T_2_-weighted images [1]. An RCC with a high intra-cystic protein content may mimic cystic pituitary adenoma. Both entities may show similar MRI characteristics (T_1_-hyperintensity and T_2_-hypointensity) which makes their differential diagnosis difficult [8]. Single-shot fast spin-echo diffusion-weighted imaging has also been tested to differentiate RCCs from other cystic lesions of pituitary fossa, but there was no significant difference observed between RCCs and cystic components of pituitary adenomas thus necessitating the need for new methods [8,9,10,11] The treatment for patients with symptomatic RCCs is mostly surgical by draining the cyst contents, including the removal of surrounding cyst wall [4,6,7]. When there is no communication with the cerebrospinal fluid, a more aggressive surgical approach of intraoperative ethanol cauterization is considered [1,12] Currently, medical professionals have no method available to unambiguously differentiate these cysts from tumors prior to surgery, and often opt for surgery to be sure of not leaving the tumor in place. Therefore, preoperative confirmation of RCCs would be of great help to them in the surgical treatment planning. Combining MRI features with in vivo magnetic resonance spectroscopy (MRS) would be valuable for the definitive diagnosis of RCCs. The goal of this study was to unravel metabolic features of RCCs using freshly resected surgical specimens by ex vivo multidimensional ^1^H NMR spectroscopy. This information can subsequently be used in the future to design an MRS diagnostic method to detect RCCs using clinical MRI scanners.

## 2. Results

**Figure 1:**
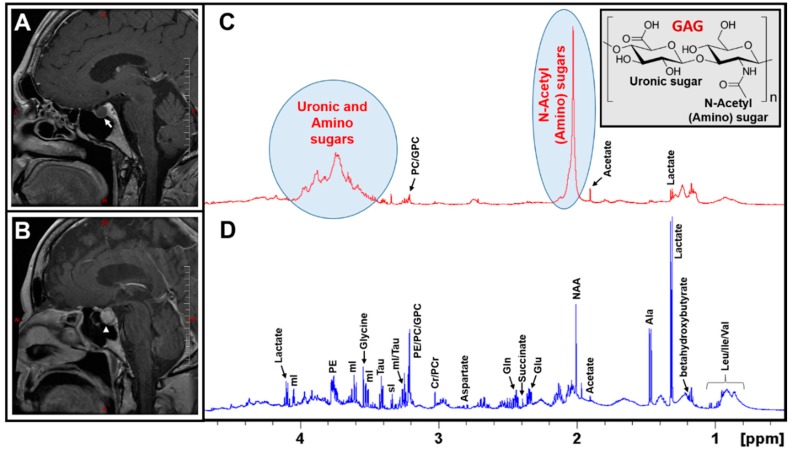
Post-contrast sagittal T_1_-weighted MR images of a Rathke’s Cleft Cyst RCC (**A**), an NF Pituitary adenoma (**B**); a thin peripheral enhancement on the post-contrast imaging in RCC is marked by a white arrow in Figure 1A, whereas the pituitary adenoma is marked with an arrow-head in Figure 1B. Typical ^1^H NMR spectra of aqueous metabolites of an RCC and an NF pituitary adenoma are shown in (**C**,**D**), respectively. It is worthwhile to note that RCCs exclusively showed the presence of N-acetyl and uronic sugars arising from glycosaminoglycans (GAGs). Also shown is the partial molecular structure of a GAG (Inset of Figure 1C).

Figure 1A,B illustrates post-contrast sagittal T_1_-weighted MR images of an RCC and a Non-functional (NF) pituitary adenoma. The MR image of the NF pituitary adenoma is characterized by the presence of a right-sided intrasellar mass with mild suprasellar extension but there is no compression of the optic chiasm or nerve (white arrow-head in Figure 1B). There was a persistent, high T_1_ signal within the mass suggesting the presence of some lipid material within the mass. The differential diagnosis could be a cystic hemorrhagic adenoma or craniopharyngioma. On the other hand, the precontrast MR image of the RCC showed a focal T_1_-hyperintense lesion but showed a thin peripheral enhancement on the postcontrast imaging with no evidence of central enhancement (white arrow in Figure 1A). Although RCCs can be suspected by MRI characteristics of pituitary lesions, the differential diagnosis also suggests a cystic pituitary adenoma.

An analysis of the metabolic composition of RCCs using MRS will play a crucial role towards a better diagnosis and, therefore, we designed this study to characterize both RCCs and NF pituitary adenoma tissue extracts using ^1^H NMR spectroscopy. Figure 1C,D show the ^1^H NMR spectra of aqueous extracts of an RCC and an NF pituitary adenoma. The ^1^H NMR spectrum of NF pituitary adenoma shows the presence of common amino acids (leucine, isoleucine, valine, alanine, glutamate, glutamine, aspartate, glycine, tyrosine, phenylalanine, and histidine), taurine, the ketone body (betahydroxybutyrate, BHB), glucose, lactate, inositols (myo (mI) and scyllo (sI)), acetate, N-acetylaspartate (NAA), succinate, creatine (Cr), phosphocreatine (PCr), and precursors for phospholipid synthesis (phosphoethanolamine (PE), choline, phosphocholine (PC), and glycerophosphocholine (GPC)) [13]. In contrast, the ^1^H NMR spectrum of an RCC predominantly showed the signals from mucopolysaccharides, which are glycosaminoglycans (GAGs) consisting of repeating units of a disaccharide molecule made up of an aminosugar (N-acetylglucosamine or N-acetylgalactosamine) and a uronic sugar (glucuronic acid) (structure shown in the inset in Figure 1C) [13,14,15]. In addition, low levels of lactate, alanine, acetate, Cr/PCr, PC/GPC, and taurine were detected in RCCs.

The presence of GAGs in RCCs was confirmed by 1D ^1^H NMR and 2D ^1^H-^1^H total correlated spectroscopy (TOCSY) experiments. In the 1D ^1^H spectrum, the characteristic ^1^H NMR signals of the N-acetyl group due to the amino sugars appear at 2.03 ppm and the broad signals in the region between 3.35 and 4.0 ppm are due to uronic sugars and other protons from amino sugars (Figure 1C).

**Figure 2:**
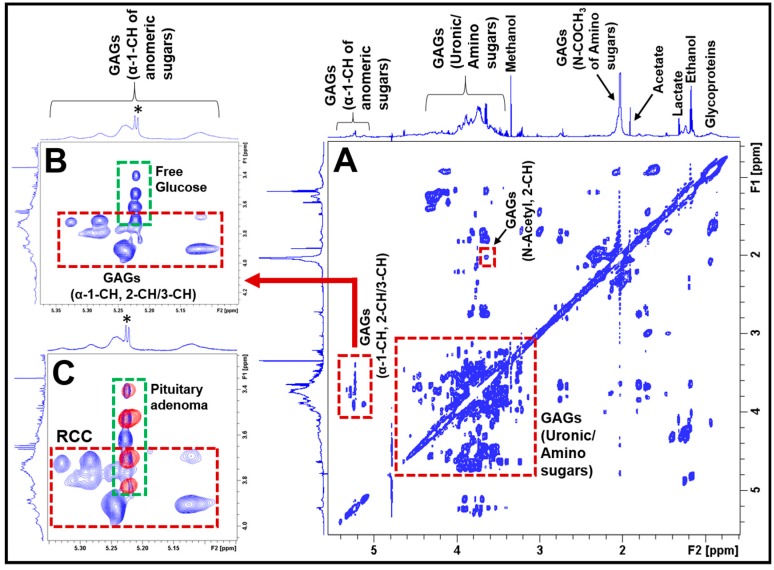
(**A**) Typical 2D ^1^H-^1^H TOCSY spectrum of aqueous extract of an RCC showing ^1^H-^1^H spin–spin coupling interactions between different sugar moieties in GAGs. The spectra on the left side and above the 2D TOCSY spectra represent 1D ^1^H NMR projections. The inset (**B**) shows the spin–spin coupling interactions between the anomeric α-1-CH and α-2-CH/α -3-CH protons of the constituent amino and uronic sugar moieties in GAGs (red box). This region also shows the presence of free glucose in RCCs (green box, the asterisk (*) represents 1D ^1^H NMR signal of free glucose). The inset (**C**) shows the overlay of anomeric regions of 2D ^1^H-^1^H TOCSY spectra of an RCC and a pituitary adenoma. The blue cross peaks are arising from the RCC (red box), whereas the red contours (green box) are from the pituitary adenoma, indicating that the blue cross peaks (in red box) are unique for RCCs.

2D NMR experiments such as ^1^H-^1^H TOCSY help in the unambiguous identification of metabolites which may be overlapping in the 1D spectra, in complex biological samples. Figure 2 shows the 2D ^1^H-^1^H TOCSY spectrum of aqueous extract of an RCC showing spin–spin coupling interactions of amino and uronic sugars in GAGs. The upper inset in Figure 2 (Figure 2B) shows the cross peaks occurring due to ^1^H spin–spin couplings between anomeric protons (α-1-CH) and 2-CH/3-CH protons of various sugar units of GAGs. The group of broad peaks (1D ^1^H NMR projection shown on the top of the TOCSY spectrum in Figure 2B) indicates the presence of mucopolysaccharides and are arising from anomeric protons (α-1-CH) of various sugar moieties of GAGs. The lower inset in Figure 2 (Figure 2C) shows the anomeric region of the ^1^H-^1^H TOCSY spectrum of an RCC extract overlaid with the same region of a pituitary adenoma extract. The blue cross peaks (inside the red box) are arising from GAGs present in RCCs, whereas the red cross peaks (in the green box) are due to the free glucose present in the pituitary adenomas. Free glucose was also found in RCC extract (blue cross peaks in the green box). Irrespective of the presence of the free glucose in both RCC and pituitary adenoma (green box in Figure 2C), the GAGs are exclusively present in RRC (the unique cross peaks in the red box, Figure 2B or Figure 2C). Thus, in the 2D TOCSY spectrum of a pituitary lesion extract, the presence of the cross peaks (shown in the red box) invariably confirms the presence of RCCs. ^1^H chemical shift values of GAGs in the current study are in agreement with the literature data [15,16,17,18,19].

**Figure 3:**
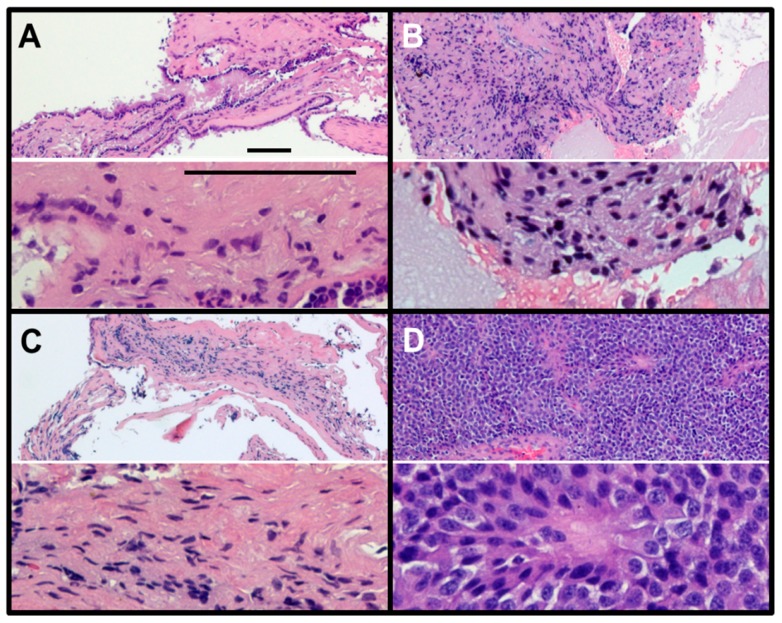
Hematoxylin and eosin (H&E) staining of RCCs (**A**–**C**) and NF pituitary adenoma (**D**). RCCs show very little nuclear staining (hematoxylin, blue) but show very large regions of hydrophobic protein (eosin, red). In contrast, the NF pituitary adenoma shows close-packed small cells with only a few regions labelled with eosin (red), the typical features of a tumor (magnification: upper slide, 10×; lower slide, 40×; scale bar = 100 µm).

Figure 3 depicts the H&E images of RCCs from three patients (Figure 3A–C) and a pituitary adenoma (Figure 3D) from an NF pituitary adenoma patient. Pituitary adenoma is characterized by the presence of monomorphous neuroendocrine cells, degenerating red blood cells and pigment-laden macrophages. On the other hand, RCCs showed features of acellular mucoid debris with intermixed eosinophilic proteinaceous globules and scattered epithelial cells embedded within the RCC material (more details in Table 1). These features of RCCs further corroborates our findings that the RCCs contain mucopolysaccharides (GAGs).

**Figure 4:**
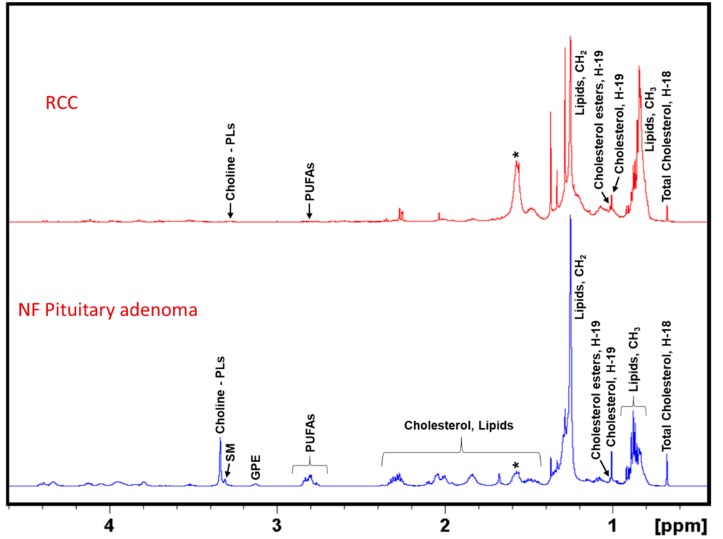
Representative ^1^H NMR spectra of lipid extracts of NF pituitary adenoma and RCC. NF Pituitary adenoma shows the presence of cholesterol, cholesterol esters, PUFAs, glycerophosphoethanolamine (GPE), choline-containing phospholipids (Choline-PLs), sphingomyelin (SM), whereas RCCs showed the presence of mostly cholesterol and low levels of cholesterol esters, PUFAs and Choline-PLs. The asterisk (*) shows the presence of residual water in the NMR solvent, CDCl_3_.

Figure 4 represents ^1^H NMR spectra of CHCl_3_ extracts of NF pituitary adenomas and RCCs showing their lipid profiles. As reported earlier, pituitary adenomas contain the common lipid metabolites such as cholesterol and cholesterol esters, polyunsaturated fatty acids (PUFAs), glycerophosphoethanolamine, Choline-PLs, sphingomyelin, and ether lipids [13]. In this study, we compared the lipid profiles of RCCs with that of NF pituitary adenomas and we found that RCCs contain very low levels of cholesterol, PUFAs and Choline-PLs. Earlier studies on RCCs reported the presence of only cholesterol [20]. In this study, we found for the first time that RCCs also contain small amounts of cholesterol esters, Choline-PLs and PUFAs.

**Figure 5:**
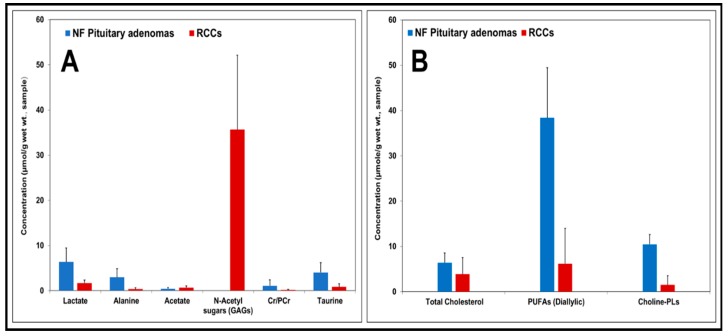
Charts showing concentrations of aqueous metabolites and lipid molecules (expressed in µmol/g, wet weight of sample, mean ± S.D.) in NF pituitary adenomas and RCCs. In comparison with pituitary adenomas, low levels of lactate, alanine, Cr/PCr, taurine and very high levels of N-acetyl sugars or GAGs were present in RCCs (**A**). Relatively lower levels of cholesterol, PUFAs and Choline-PLs were present in RCCs (**B**).

In addition, we quantified the levels of both aqueous metabolites and lipids molecules in RCCs and NF pituitary adenomas. Figure 5A shows concentrations of aqueous metabolites (µmol/g, wet weight of sample, mean ± S.D.) in NF pituitary adenomas and RCCs. Metabolite concentrations in the NF pituitary adenoma group were compared with those in RCCs. The levels of lactate, alanine, Cr, PCr, and taurine were decreased in RCCs when compared to NF pituitary tumors. One of the striking differences we observed in RCCs was that the RCCs showed the presence of very high levels of N-acetyl sugars (35.68 ± 16.43 µmol/g) arising from GAGs which were absent in NF pituitary adenomas. Figure 5B shows concentrations of lipid molecules (µmol/g, wet weight of sample, mean ± S.D.) in NF pituitary adenomas and RCCs. The levels of cholesterol, PUFAs and Choline-PLs were lower in RCCs compared to the NF pituitary tumors.

## 3. Discussion

In the current study, we have determined the metabolic composition of RCCs and compared it with that of NF pituitary adenomas by 1D and 2D ^1^H NMR spectroscopy and identified unique metabolites which can be used to differentiate RCCs from the NF pituitary adenomas. We found that the RCCs exclusively contained GAGs which were absent in NF pituitary adenomas. The presence of GAGs in RCCs can be confirmed by looking at the broad ^1^H NMR signals of the N-acetyl group resonating at 2.03 ppm or uronic sugars signals in the region 3.35–4.0 ppm (Figure 1C) or anomeric sugar signals in the region 5.05–5.35 ppm (Figure 2B). Recently, we also conducted an extensive NMR-based metabolomics study on various immunohistochemical subtypes of pituitary adenomas [13], and we found that none of these subtypes including the functional pituitary adenomas (PRL/ACTH/LH-FSH and GH secreting) showed the presence of GAGs [13]. Given GAGs are exclusively detected in RCCs, they may be used as metabolic markers for the detection of RCCs.

This observation will have far-reaching applications in developing a diagnostic method for the preoperative confirmation of RCCs. Currently, medical professionals rely on intraoperative histopathological frozen section analysis of pituitary lesions to confirm the diagnosis of RCCs before they proceed further towards the completion of the surgery [11,20]. Therefore, preoperative confirmation of RCCs would be of help to medical professionals in surgical treatment planning. Our current ^1^H NMR characterization of RCCs would form a basis for its translation into an in vivo MRS methodology using clinical MRI scanners. In vivo ^1^H MRS of suprasellar tumors has been tested [21,22] and the information obtained increased the diagnosis of suprasellar tumors by 25% when compared to MRI alone [21]. Therefore, combined use of both MRI and MRS will be of great advantage in the diagnosis of pituitary lesions.

Pituitary adenoma contains the neuronal osmolyte NAA whose ^1^H NMR signal appears at 2.01 ppm (Figure 1D), which may interfere with the N-acetyl signal of GAGs (at 2.03 ppm, Figure 1C) in RCCs during an in vivo ^1^H MRS acquisition. NAA signal can be suppressed by applying spatial saturation pulses/bands (available with the modern MRI/MRS scanners) around the spectroscopic voxel during data collection. Moreover, NAA yields a very narrow peak (full width at half maximum, FWHM = 1.40 Hz) compared to the N-acetyl peak of GAGs (FWHM = 8.55 Hz) as shown in Figure 1C,D, its presence can be tested by collecting ^1^H MRS data with a longer echo-time (TE) than the TE used for detecting N-acetyl sugars. It is also worthwhile to note that both pituitary adenomas and RCCs contained the free glucose, but only RCCs showed the presence of multiple signals arising from anomeric protons of the sugar units of GAGs (Figure 2C). These multiple signals are unique signatures showing the presence of GAGs and can serve as metabolic markers for the diagnosis of RCCs (Figure 2B,C). Localized two-dimensional (2D) correlated spectroscopy (L-COSY) technology has been routinely used in the non-invasive diagnostics of various neurological diseases including malignant brain tumors that offers superior sensitivity in detecting various metabolites compared to conventional 1D MRS [23]. This technology may allow us to detect GAGs in vivo similar to our ex vivo 2D TOCSY results, as described in the results section (Figure 2).

To the best of our knowledge, there are no reports on the metabolomics of cystic pituitary adenomas. As we demonstrated in this study, only RCCs contained high concentrations of GAGs (35.68 ± 16.43 µmol/g). Given the partial cystic nature of cystic pituitary adenomas, low levels of GAGs may also be present in them that can be detected by ^1^H MRS methods described in this study. Although we are continuously enrolling pituitary tumor patients, we have not had yet a single cystic pituitary adenoma subject for the treatment. Our IRB protocol is open to enroll all subtypes of pituitary tumors and, therefore, we will investigate the metabolomics of cystic pituitary adenomas when patient specimens become available.

## 4. Materials and Methods

### 4.1. Patients

RCC specimens were collected (and snap-frozen) from five patients undergoing endoscopic transsphenoidal selective lesionectomies at the Houston Methodist Hospital who were suspected of having an RCC or a cystic pituitary adenoma. ^1^H NMR data from patients with NF pituitary adenomas (n = 5), from our recent study, were used for comparison [13]. These NF pituitary adenomas were solid tumors and did not contain any cystic component. We chose NF pituitary tumors because they do not secrete any hormones as RCCs. All diagnoses were confirmed with clinical histopathological analysis. The study was approved by the Institutional Review Board of Houston Methodist Hospital and Research Institute. Informed consent was obtained from each patient before the surgery. Patients’ characteristics along with clinical presentations, MRI findings, and details on immunohistochemistry (IHC) have been provided in Table 1.

### 4.2. Immunohistochemistry (IHC)

Immunohistochemical analysis of the RCCs and pituitary adenomas was performed at the Houston Methodist Hospital Histology Laboratory. Intraoperative RCCs and pituitary adenoma specimens were stained for hematoxylin and eosin (H&E). In addition, the pituitary adenomas were also stained for prolactin (PRL), adrenocorticotropic (ACTH), growth (GH), luteinizing (LH), follicle-stimulating (FSH), and thyroid-stimulating (TSH) hormones including Ki-67 (MIB-1) proliferation index.

### 4.3. Chemicals

The solvents—methanol, chloroform, and chloroform-d (CDCl_3_) were purchased from Millipore Sigma (St. Louis, MO, USA). Deuterated chemicals (deuterium oxide, D_2_O; deuterium chloride, DCl; and sodium deuteroxide, NaOD) were commercially available and purchased from Cambridge isotope laboratories (Tewksbury, MA, USA). Internal standard for metabolite quantification, 3-(trimethylsilyl)-1-propane sulfonic acid-*d*_6_ sodium salt (DSS-*d*_6_) was purchased from Chenomx Inc. (Edmonton, AB, Canada).

### 4.4. Methanol–Chloroform Extraction

RCC specimens were extracted using methanol–chloroform solvent mixture as described in our recent publication [13]. Briefly, the RCC specimens were weighed (14–50 mg) and transferred to tubes containing zirconium oxide (ZrO_2_) beads (Benchmark Scientific, Edison, NJ, USA). To the tubes containing the specimens, 0.5 mL of methanol:chloroform solvent mixture (2:1 ratio, v/v, 4 °C) was added and the tubes were spun at 4000 rpm for 2 min using a tissue homogenizer (Benchmark Scientific, Edison, NJ, USA). The above step was repeated two more times, and an additional 0.25 mL each of chloroform and Millipore water were mixed with the extract homogenate and were spun further for 2 min. The methanol and chloroform layers were isolated carefully by centrifugation at 10,000× *g* for 10 min and solvents were dried under vacuum. Residues from the methanol layer (water soluble) were dissolved in 180 µL of D_2_O containing 1.0 mM DSS-*d*_6_ as an internal standard, and the final pH was adjusted to 7.4 ± 0.05. Whereas, lyophilized residues of chloroform layer of the extracts were dissolved in 180 µL CDCl_3_ containing 0.05% tetramethylsilane (TMS) as the internal standard for quantification of lipid metabolites.

### 4.5. 1D and 2D ^1^H NMR Experiments

1D ^1^H and 2D ^1^H-^1^H Total Correlated Spectroscopy (TOCSY) NMR data of RCCs were collected on a Bruker 600 MHz spectrometer (Bruker Biospin, Billerica, MA, USA). ^1^H NMR spectra of RCC extract samples in 3 mm NMR tubes were obtained by using nuclear Overhauser effect (NOESY) pulse sequence with water pre-saturation (for aqueous-methanol extracts) and a single 30° radiofrequency (RF) pulse excitation sequence was used for CHCl_3_ extracts. 1D NMR data were collected with the following acquisition parameters: number of scans = 128; 90° RF pulse = 10 µs; number of data points = 64 k; relaxation delay = 5.0 s; spectral width = 9615 Hz; acquisition time = 2.73 s; mixing time for NOESY = 100 ms; and line broadening window function = 0.3 Hz. DSS-d_6_ and TMS were used as internal chemical shift and concentration reference standards in the ^1^H spectra of methanol and chloroform extract samples, respectively. Bruker TopSpin 3.5 software was used for NMR data processing and the determination of the peak areas of the ^1^H NMR resonances. 2D TOCSY experiment was performed for the spectral assignments of some of the overlapping proton signals in the 1D spectrum. The parameters used for acquiring 2D TOCSY NMR data were: spectral width = 9590.8 Hz in both dimensions; time domain data points = 4096; t_1_ increments = 512; relaxation delay = 2 s and number of transients = 40. RF spin lock was applied for 80 ms in TOCSY experiments.

### 4.6. Identification and Quantification of Metabolites

The aqueous metabolites in the aqueous-methanol layer and lipid molecules in the chloroform layer were identified by analyzing 1D ^1^H and 2D ^1^H-^1^H TOCSY NMR spectra and also by comparing the chemical shifts reported in our recent publications [13,14]. Aqueous metabolites and lipid molecules from RCCs were quantified using the following equation:

(1) $$\begin{array}{l} \mathrm{Concentration}\;\left(\frac{µ\mathrm{mol}}{g}\right)\\ \;=\frac{\#\mathrm{protons}\;\mathrm{in}\;\mathrm{standard}}{\#\mathrm{protons}\;\mathrm{in}\;\mathrm{analyte}}\times \frac{\mathrm{peak}\;\mathrm{area}\;\mathrm{of}\;\mathrm{analyte}}{\mathrm{peak}\;\mathrm{area}\;\mathrm{of}\;\mathrm{standard}}\\ \;\times \frac{\mathrm{volume}\;\mathrm{of}\;\mathrm{the}\;\mathrm{extract}\;\left(\mathrm{mL}\right)}{\mathrm{mass}\;\mathrm{of}\;\mathrm{the}\;\mathrm{wet}\;\mathrm{tissue}\;\left(g\right)}\times \mathrm{concentration}\;\mathrm{of}\;\mathrm{standard}\;\left(\mathrm{mM}\right) \end{array}$$

For aqueous metabolites: number of protons contributing to the internal standard (DSS-d_6_) was 9; for lactate, alanine, acetate, N-acetyl sugars, Cr/PCr was 3; and for taurine was 2. Similarly, for lipid molecules: number of protons contributing to the internal standard (TMS) was 9; for cholesterol was 3, PUFAs was 2 and for Choline-PLs was 9.

## 5. Conclusions

Here, we have shown the metabolic composition of surgically resected RCCs from five patients using ^1^H NMR spectroscopy. RCCs exclusively contain GAGs, glycosaminoglycans, which are not detected in pituitary adenomas. GAGs can serve as metabolic marker for the detection of RCCs. MRI is the mainstay of diagnosis of pituitary gland pathology and recent developments in pituitary imaging have provided valuable information in the diagnosis and pre-surgical planning of pituitary adenomas. MRS is a non-invasive metabolic imaging technique similar to MRI which provides biochemical information of healthy/pathological tissue, thereby facilitating a better diagnosis. In vivo ^1^H MRS of suprasellar masses or tumors is feasible using clinical MRI/MRS scanners [21,22,24], and this technique can be augmented to the pituitary MRI protocol. Based on our current data, in vivo MRS methods can be designed to detect GAGs in patients who are suspected to have RCCs. Combining in vivo MRS and MRI will help in the unambiguous detection of GAGs in patients with RCCs, and help in the preoperative diagnosis of RCCs. The current results pave the way for the implementation of localized multidimensional in vivo MRS techniques in the routine radiological diagnosis of RCCs including their differentiation from the pituitary adenomas. Further studies are needed to characterize the cystic pituitary adenomas and their differentiation from RCCs.

## Figures and Tables

**Table 1:** Characteristics of patients with NF pituitary adenoma and RCC.

Patient	Age (y)/Sex	Clinical Characteristics	Hormone Panel	MRI Findings (Tumor Size)	Indications for Surgery	Immunohistochemistry (IHC)
1	46/F	Blurry vision, fullness in ears	LH = 11.1 FSH = 16.9 PRL = 9.0 GH = <0.05 TSH = NA ACTH = NA	Showed a pituitary mass. (1.4 × 1.5 × 1.7 cm^3^)	Since serum PRL was normal, medical therapy was not an option, indicating surgery to resect tumor mass.	Exfoliation of neuroendocrine appearing cells, effacement of normal adenohypophyseal architecture and replacement by neuroendocrine appearing cells; tumor cells negative for PRL, LH, FSH, ACTH, TSH, and GH. MIB-1 = 1%
2	37/M	Visual field deficit, stretch marks on stomach and back, weight gain suggesting Cushing’s disease.	LH = 2.1 FSH = 2.4 PRL = 7.6 GH = 0.1 TSH = NA **ACTH = 110.0** Cortisol (urine/24 h) = 13.1 µg/L	Pituitary macroadenoma with suprasellar extension, (3.9 × 2.3 × 2.4 cm^3^)	Tumor mass was compressing optic chiasm and nerves.	Same as above. MIB-1 = 3%
3	39/F	Pituitary tumor recurrence, previously underwent surgery 2 times.	LH = 4.8 FSH = 9.7 PRL = 17 GH = 0.31 TSH = 1.31 ACTH = 22.8 free T4 index = 1.7	A mass in the left side of sella, protruding into suprasellar region. (1.0 × 1.0 × 0.8 cm^3^)	Tumor mass was protruding into the suprasellar region with mass effect on the optic nerves.	Same as above. MIB-1 = 1%
4	42/M	Dizziness, headache, visual field disturbance	LH = 3.9 FSH = 4.5 PRL = 6.6 GH = 170.0 TSH = 0.15 ACTH = 41.2	A suprasellar mass. (1.8 × 2.1 × 1.3 cm^3^)	Tumor mass was uplifting the optic chiasm and prechiasmatic optic nerve.	Same as above. MIB-1 = 1%
5	75/F	Dizziness, forgetfulness, blurry vision	LH = 2.1 FSH = 3.9 PRL = 11.0 GH = 0.07 TSH = 0.42 **ACTH = 53.5** Cortisol (urine/24h) = 27.0 µg/L	Pituitary mass in the anterior aspect of the sella, hyperintense on T_1_-weighted image. (1.0 × 1.4 × 1.0 cm^3^)	Surgery was recommended due to the size of the tumor and proximity to/possible compression of the optic apparatus.	Monomorphous expansion of neuroendocrine cells, loss of normal acinar architecture, tumor cells negative for PRL, LH, FSH, ACTH, TSH, and GH. MIB-1 = 1%
6	58/M	Fatigue with dizziness, nausea and vomiting, hyponatremia and hypokalemia.	LH = 0.3 FSH = 2.0 PRL = 10.2 GH = 0.1 TSH = 3.41 ACTH = 5	An intrasellar mass with suprasellar extension. Hyperintense on pre-contrast T_1_-weighted image. (1.0 × 1.2 × 1.0 cm^3^)	Tumor mass was abutting the optic chiasm. The tumor mass was hyperintense on T_1_-image suggesting hemorrhage, and pan hypopituitarism.	Acellular mucoid debris with intermixed eosinophilic proteinaceous globules consistent with cyst contents. Cyst wall showed the presence of ciliated epithelium consistent with RCC. No features of pituitary adenoma were identified.
7	38/F	Dizziness, right-sided facial numbness and headache. History of thyroid cancer currently on thyroid supplementation.	LH = 7.0 FSH = 6.7 PRL = 10.6 GH = 0.2 TSH = 0.02 ACTH = 11.0	A lesion on the right side of the pituitary gland, bulging into the suprasellar cistern without mass effect Bright on T_2_-weighted image. (0.9 × 0.7 × 0.9 cm^3^)	The lesion was extended into the suprasellar cistern and abutted the optic nerve.	Same as above, findings diagnostic of RCC.
8	25/M	Dizziness	LH = 3.2 FSH = 2.3 PRL= 5.7 GH = 0.1 TSH = 0.92 ACTH = NA	Intrasellar mass which is cystic in nature. (0.4 × 0.5 × 0.35 cm^3^)	Since serum PRL was normal, medical therapy was not indicated, and the surgery was recommended.	Proteinaceous debris and fibrotic tissue with rare ciliated cells compatible with RCC. No features of pituitary adenoma were identified.
9	45/F	Prolactin secreting pituitary lesion was suspected.	LH = 10 FSH = 0.3 **PRL = 55.05** ^#^ GH = NA TSH = 2.05 ACTH = NA	MRI showed a large pituitary mass bulging up into the suprasellar cistern. (1.1 × 1.2 × 2.0 cm^3^)	Surgery was recommended due to the presence of large pituitary mass which was extending into suprasellar cistern and compressing the optic apparatus.	Acellular debris with scattered epithelial cells consistent with RCC. No features of pituitary adenoma were identified.
10	25/F	Irregular menstrual cycle, weight gain, prolactin secreting pituitary lesion was suspected.	LH = 5.8 FSH = 2.1 **PRL = 26.9** ^#^ GH = 0.1 TSH = 1.80 ACTH = 20.8	MRI showed a pituitary lesion, differential hypo enhancement in the posterior aspect of the pituitary gland. (0.5 × 1.0 × 0.5 cm^3^)	Follow up blood work confirmed that serum PRL was normal (22.4 ng/mL), and surgical excision of the lesion was recommended.	Proteinaceous debris with bland degenerated cells compatible with RCC. No features of pituitary adenoma were identified.

(ACTH, adrenocorticotropic hormone; FSH, follicle stimulating hormone; GH, growth hormone; IHC, Immunohistochemistry; LH, luteinizing hormone; NA; not available; PRL, prolactin; TSH, thyroid stimulating hormone; (Reference ranges, FSH: 1.6–8.0 mIU/mL; GH: ≤7.1 ng/mL; LH: 1.5–9.3 mIU/mL; PRL: 2–23 ng/mL; ACTH: 6–25 pg/mL; Cortisol (urine/24 h) = 0–50 µg/24 h; TSH: 0.27–5.0 µIU/mL); Hormone panel: Hormones which were elevated (>normal range) have be given in bold letters; #, Due to elevated levels of PRL, these patients were treated with Cabergoline; Tumor size was measured in the anterior-posterior (AP), transverse (T) and craniocaudal (CC) dimensions.
